# Supplementary material for: IgStrand: A universal residue numbering scheme for the immunoglobulin-fold (Ig-fold) to study Ig-proteomes and Ig-interactomes
Source: PLoS Comput Biol. 2025 Apr 14;21(4):e1012813. doi: 10.1371/journal.pcbi.1012813 (PMC12051499; doi:10.1371/journal.pcbi.1012813)
Supplement: S1 Text — Constructed with BLOSUM 62 with the Score of Optimally-Extended Block method. Fig B. F strand Tyr (igs# 8548 or 8546) is highly conserved across many eukaryotic and bacterial Ig-like domains despite topo-structural variations in the fold. This Tyr appears to play similar roles by interacting with the EF loop, and may provide insights into the evolution of a number of proteins sharing the Ig-fold. It certainly points toward a key structural residue. Fig C. Tertiary Intrachain interfaces in the Horseshoe superdomain formed by the four N-terminal residues in Ig-chains. This superdomain allows a certain level of plasticity observed in comparing contactin-2 and DSCAM. The RMSD is 4.9Å. A) Contactin-2 Horseshoe (Pdbid 8A0Y) shows an Ig1-Ig4 and a Ig2-Ig3 antiparallel interfaces. B) Ig1-Ig4 interactome in contactin-2. C) Ig2-Ig3 interactome in contactin-2. D) Ig1-Ig2 interactome in contactin-2. E) Ig3-Ig4 interactome in contactin-2. F) Ig1-Ig4 interactome in DSCAM. G) Ig2-Ig3 interactome in DSCAM. H) Ig1-Ig2 interactome in DSCAM. I) Ig3-Ig4 interactome in DSCAM. J) Common interactions in Ig1-Ig4. This pairwise interactome is more conserved than Ig2-Ig3 that is more plastic. K) Common interactions in Ig3-Ig4.Fig D. Clustering Ig templates using MEGA11. The graph shows the distances between templates. The corresponding TM-score is equal to 1 - distance.Table A. Number of Heavy chain - Light chain contacts in VH:VL and CH1:CL interfaces in Fabs bound to SARS-CoV2 antigens or to a diverse set of antigens and present to more than 70% (or 90%) of the Fabs in each dataset.Table B. VH:VL interactions of Fabs binding diverse antigens (70% cutoff).Red numbers represent symmetric contacts. Shaded cells represent five highly conserved contacts (90% cutoff) shared between the SARS-CoV-2 antigen binding dataset (Table 2 in manuscript) and this diverse antigen binding dataset. Bold contacts represent highly conserved hydrogen bonding contacts. Underlined contacts represent those that hav [file pcbi.1012813.s001.docx]

A universal residue numbering scheme for the Immunoglobulin-fold (Ig-fold) to study Ig-Proteomes and Ig-Interactomes

Caesar Tawfeeq^1^, Jiyao Wang^2^, Umesh Khaniya^3^, Thomas Madej^2^, James Song^2^, Ravinder Abrol^1*^, and Philippe Youkharibache^3*^.

^1^Department of Chemistry and Biochemistry, California State University Northridge, Northridge, California, United States of America

^2^National Center for Biotechnology Information, National Library of Medicine, National Institutes of Health, Bethesda, Maryland, United States of America

^3^Cancer Data Science Laboratory, Center for Cancer Research, National Cancer Institute, National Institutes of Health, Bethesda, Maryland, United States of America.

^*^[abrol@csun.edu](mailto:abrol@csun.edu) (RA) and [philippe.youkharibache@nih.gov](mailto:philippe.youkharibache@nih.gov) (PY)

SUPPLEMENTARY INFORMATION

**
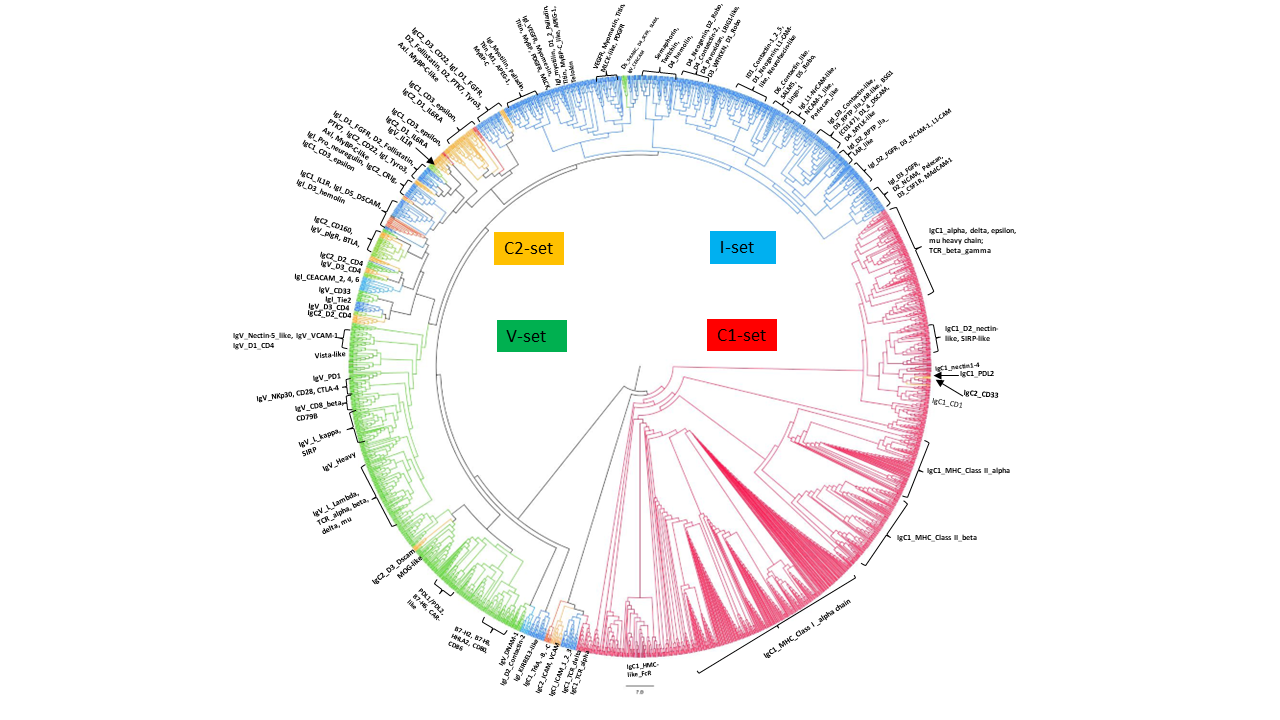
**

**Fig A. CDD IgSF Tree**. Constructed with BLOSUM 62 with the Score of Optimally-Extended Block method.

| **A**  **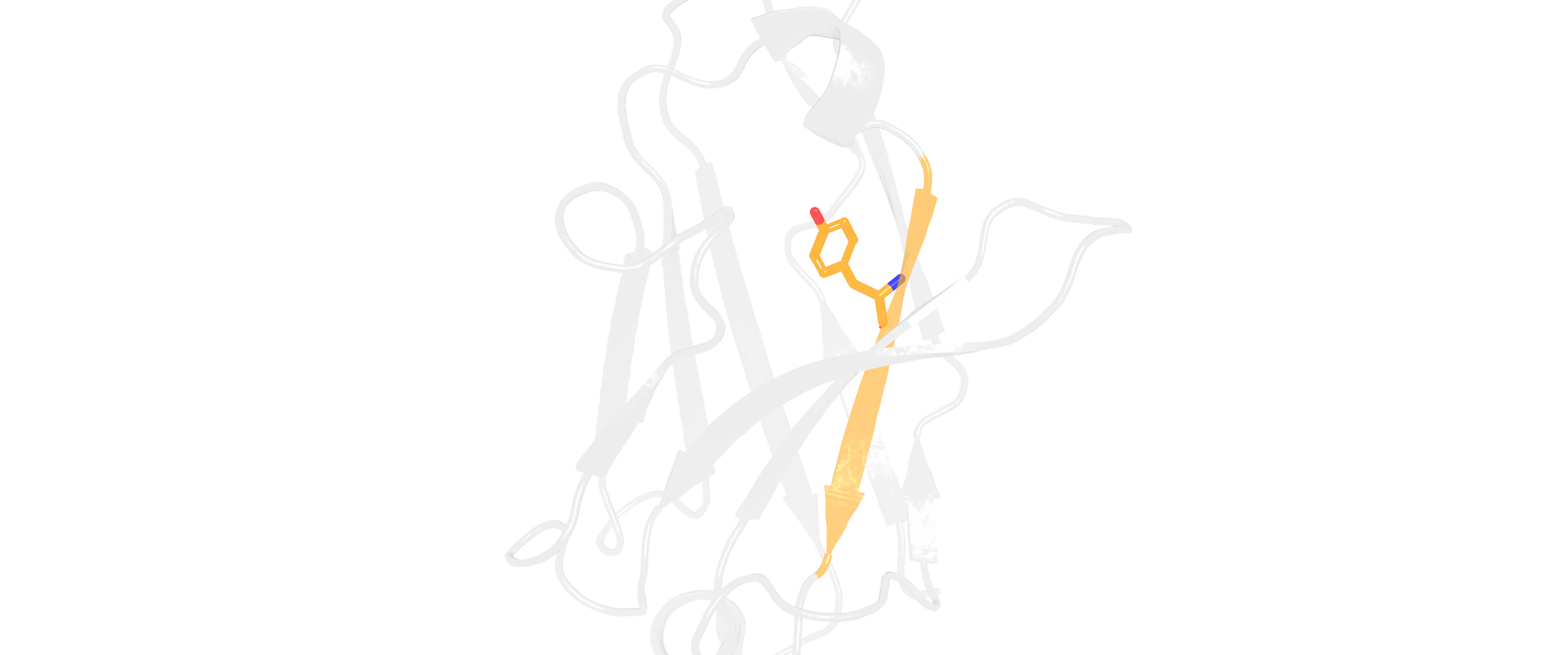**  **VH (Classical)**  **(5ESV)** | **B**  **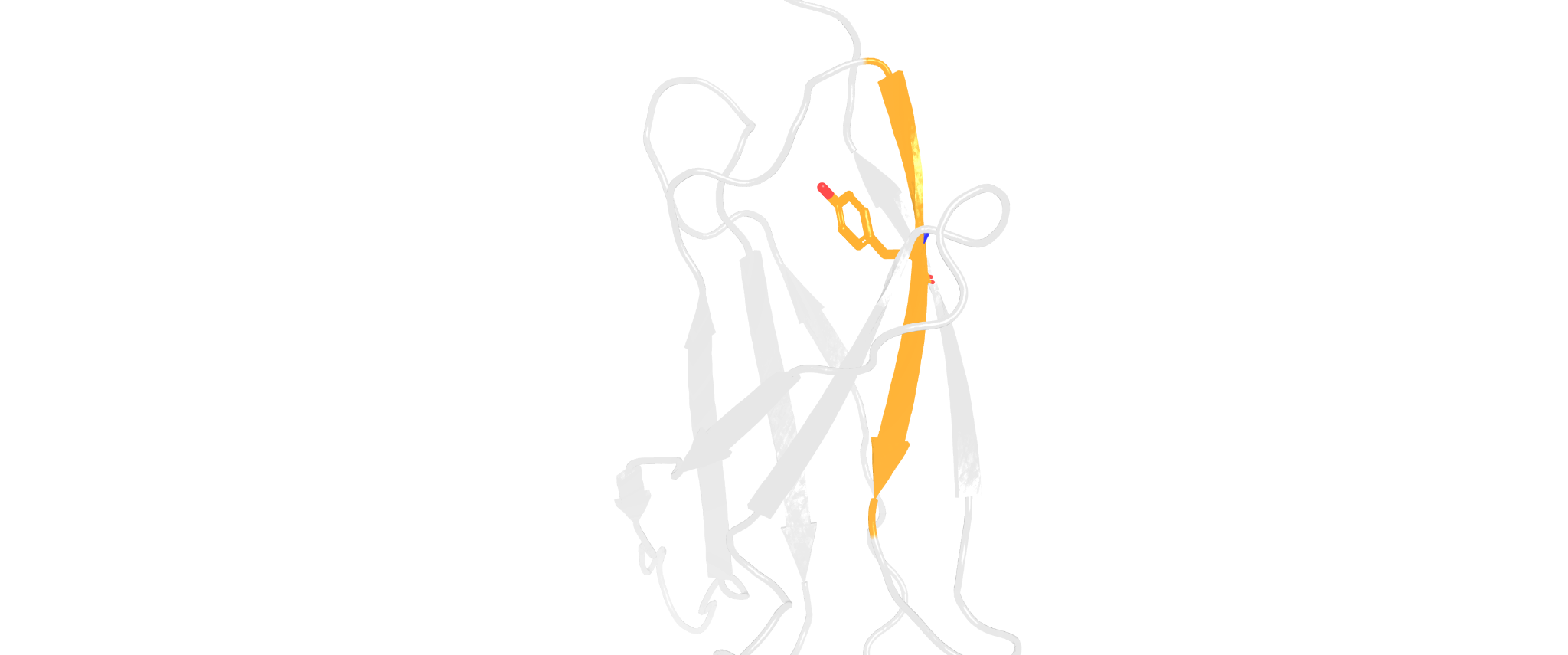**  **Purple Acid Phosphatase**  **(1XZW, plant)** | **C**  **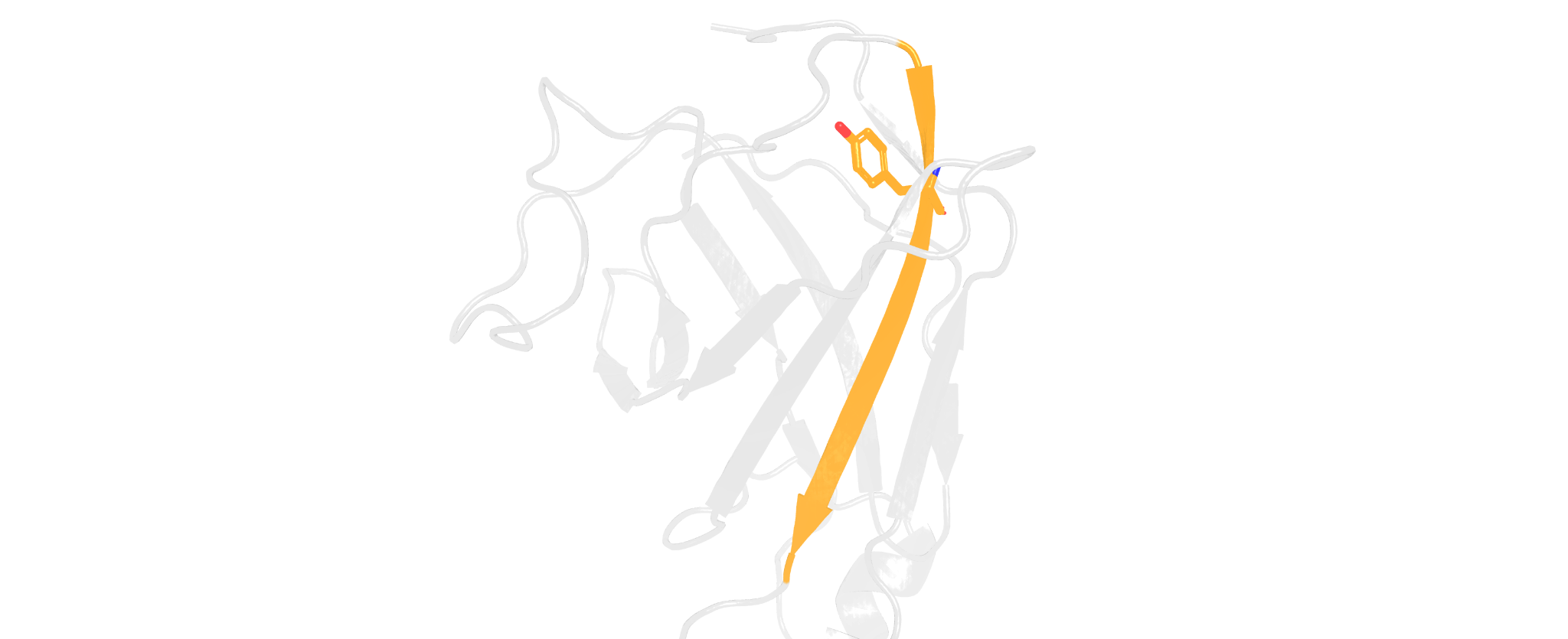**  **TP34**  **(2O6C, bacteria)** | **D**  **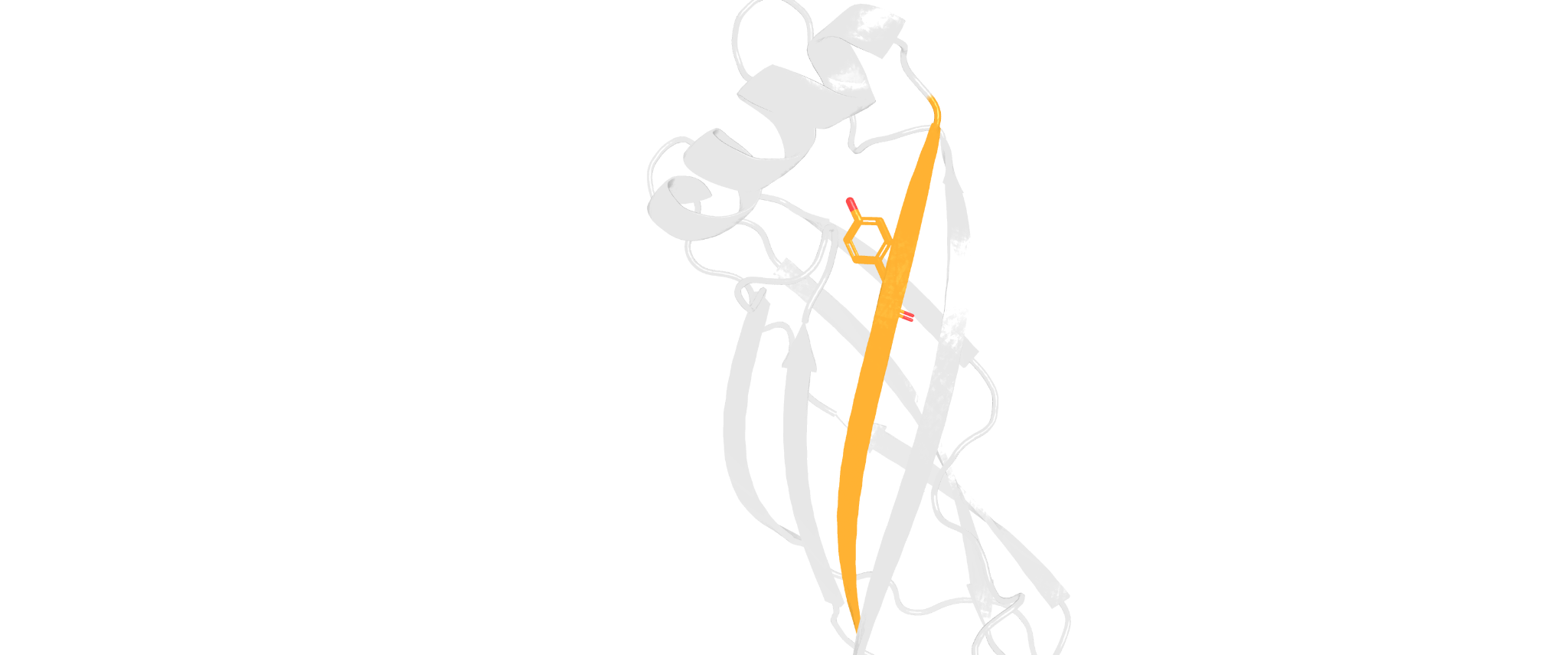**  **LEA14**  **(1XO8, plant)** |
| --- | --- | --- | --- |
| **E**  **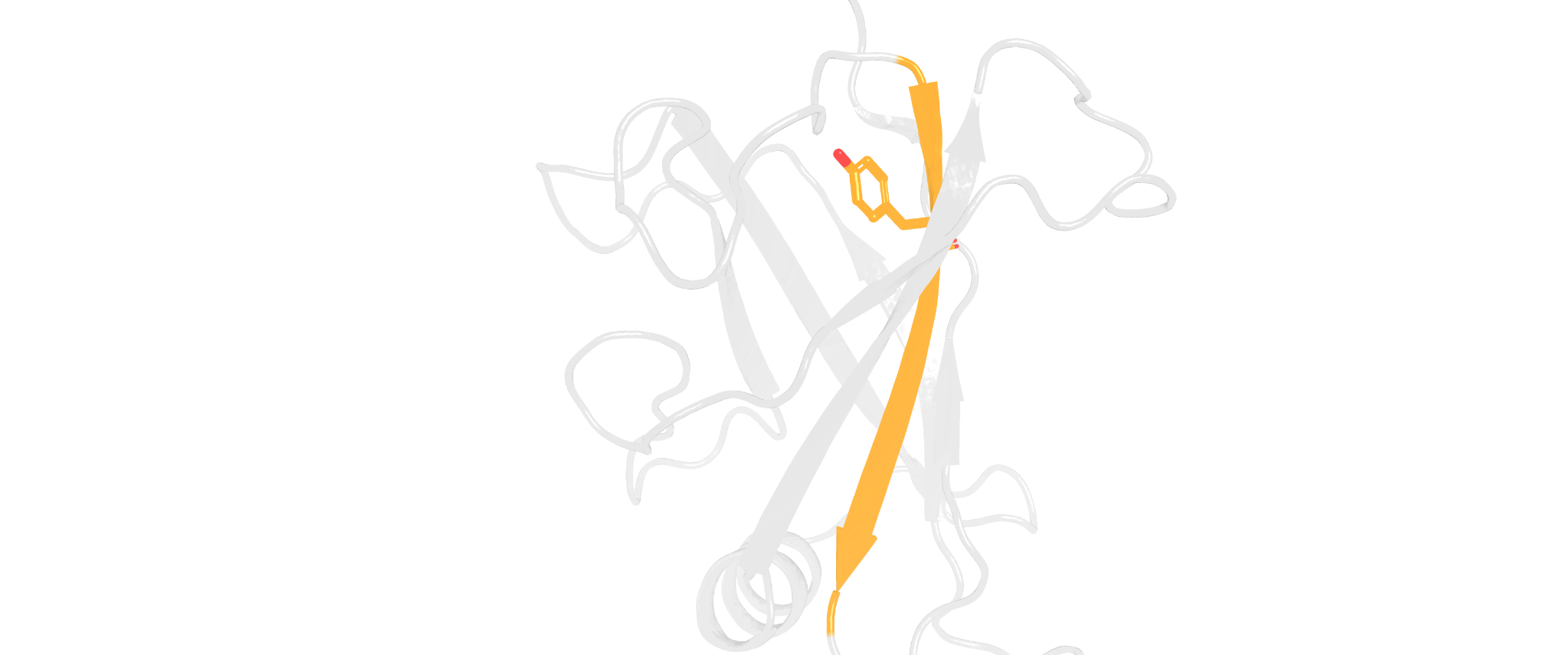**  **TP47**  **(1O75, bacteria)** | **F**  **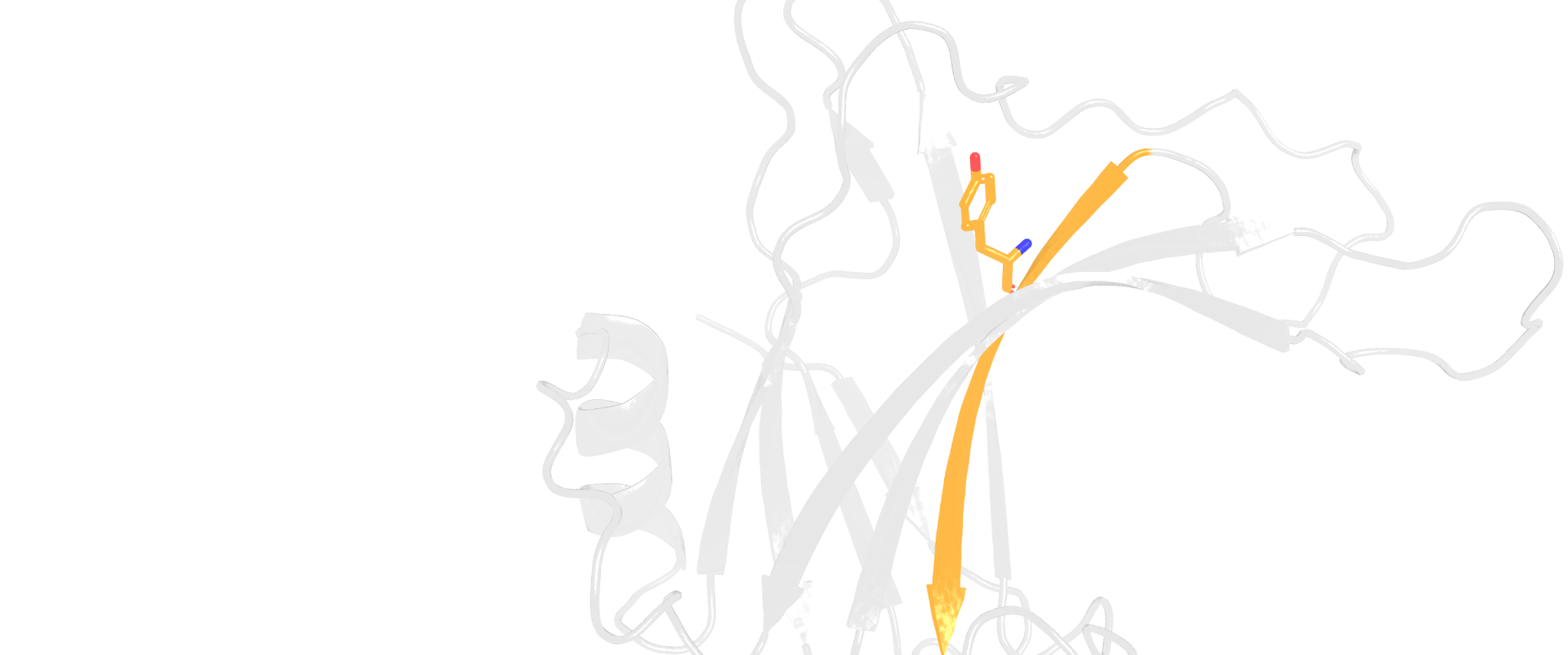**  **beta-Arrestin**  **(4JQI, rat)** | **G**  **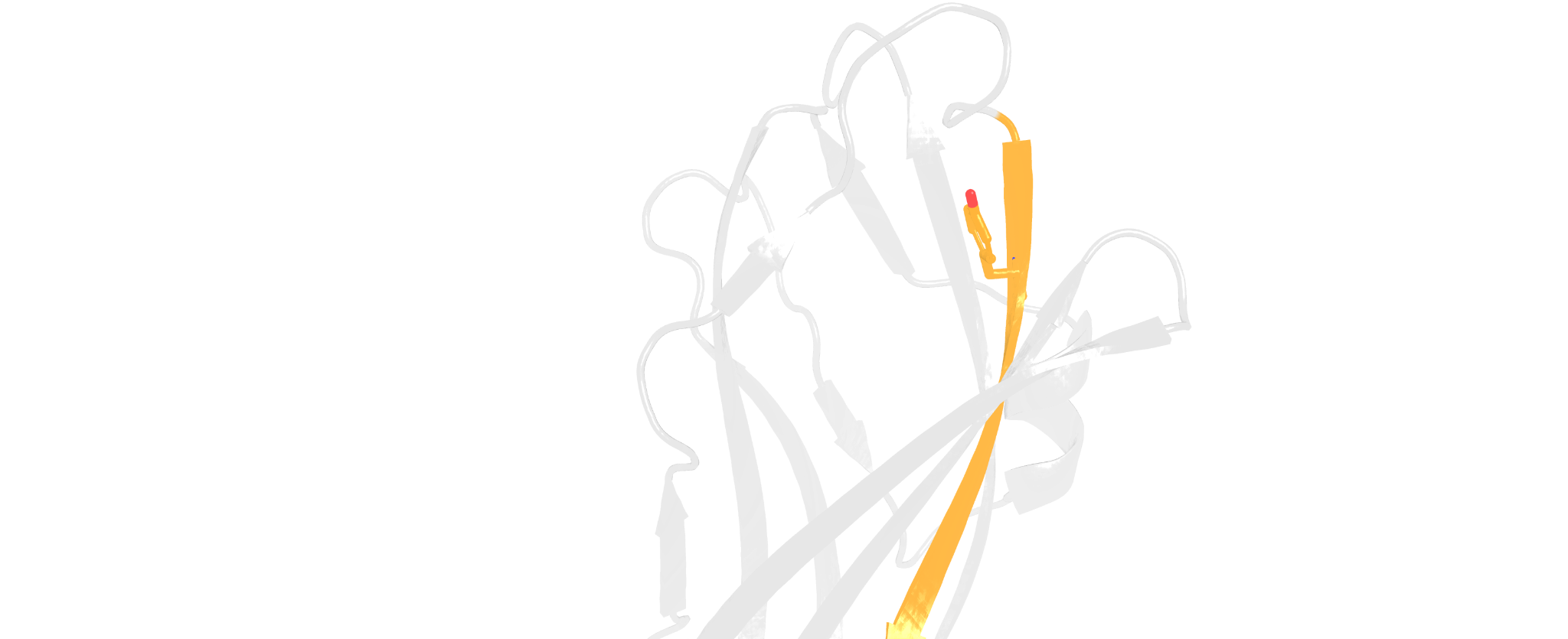**  **rhoGDP Dissociation Inhibitor**  **(1CC0, human)** | **H**  **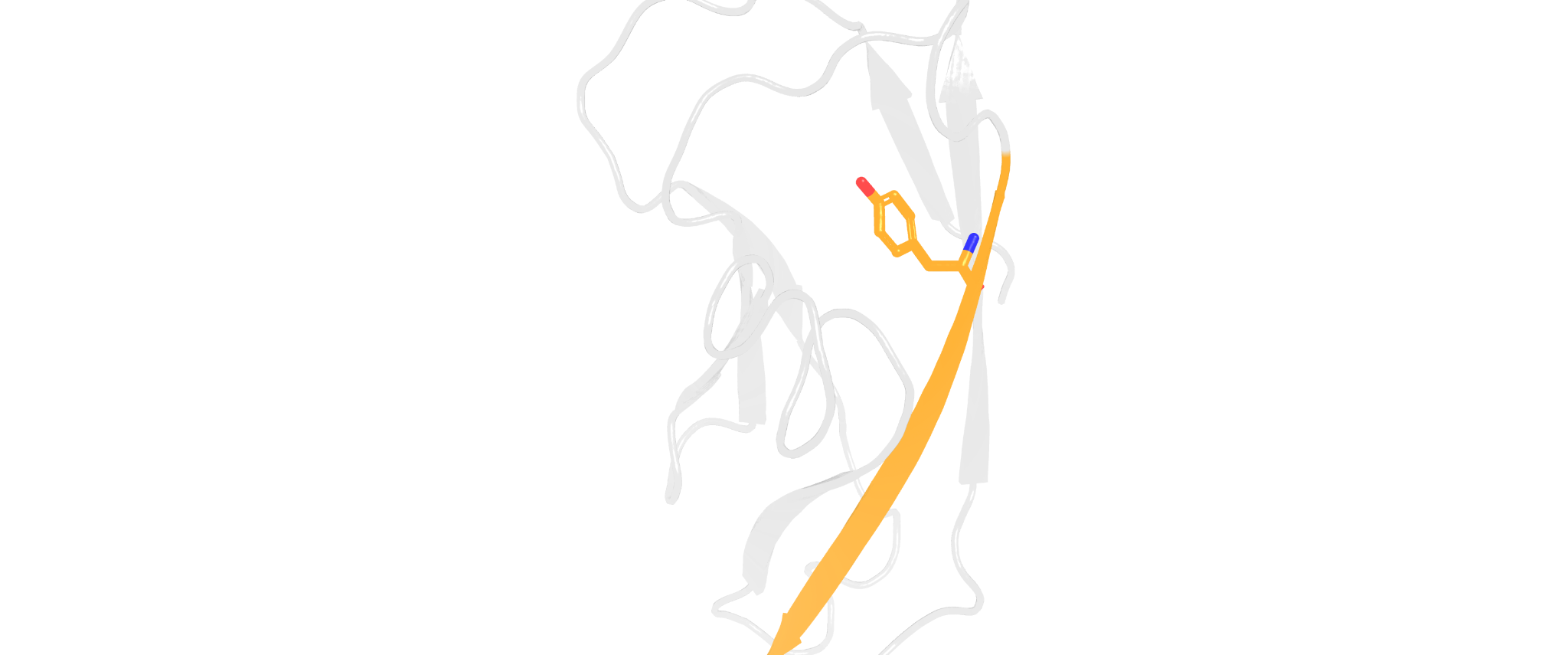**  **E-Cadherin**  **(4ZT1, human)** |
| **I**  **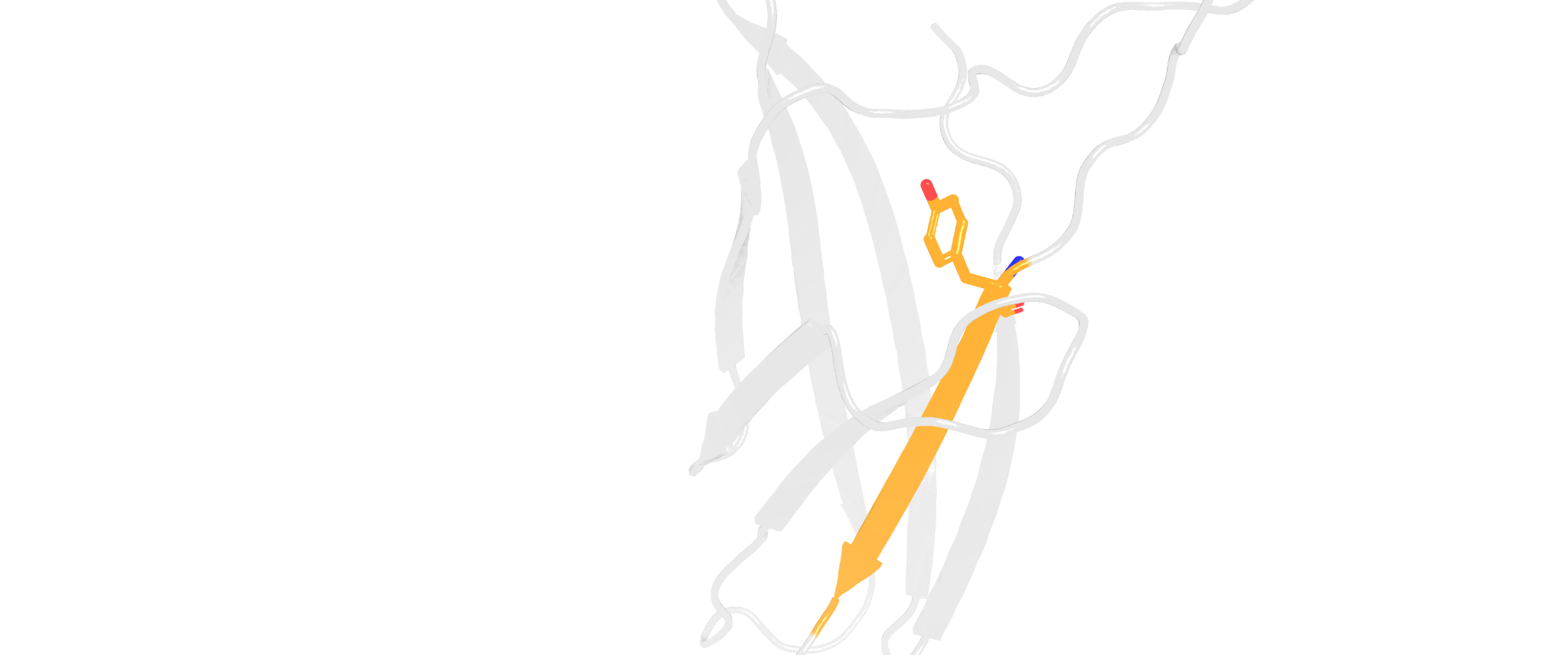**  **Glucuronidase**  **(3HN3, human)** | **J**  **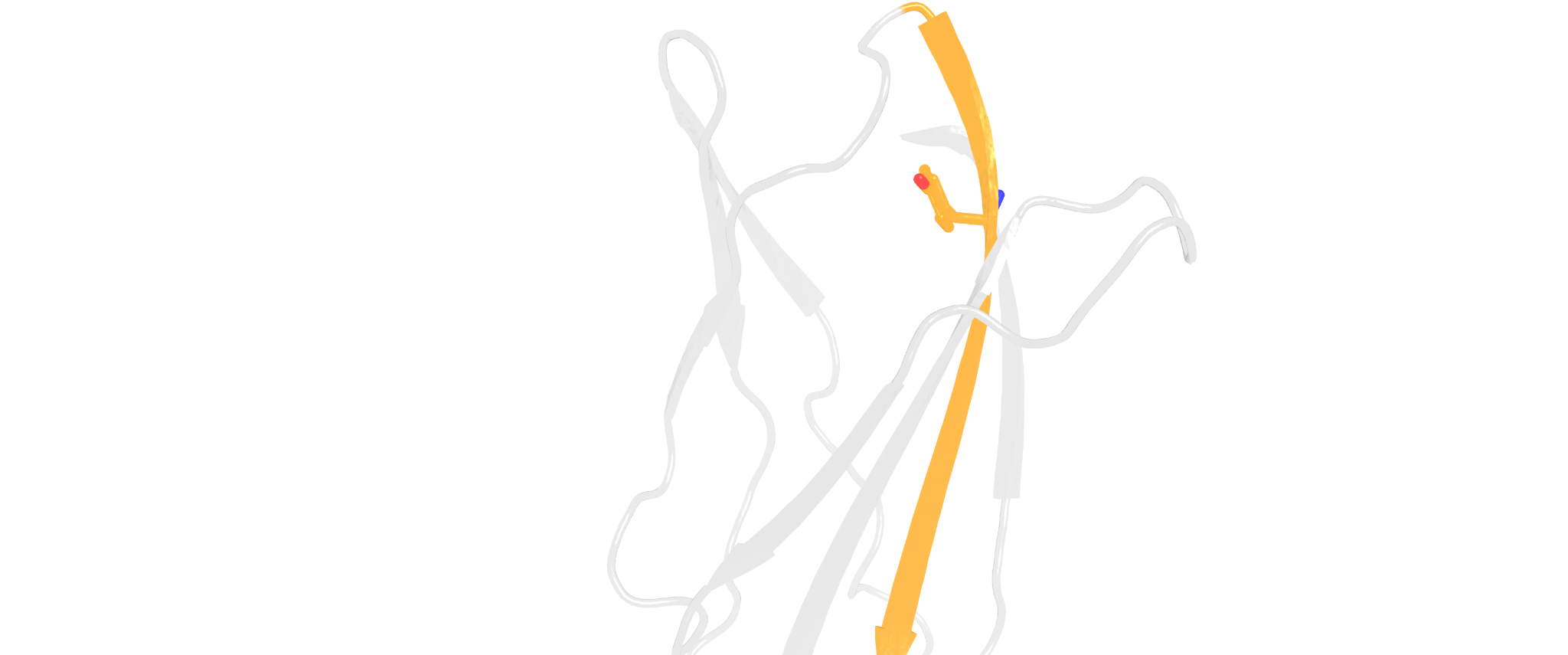**  **Fibronectin**  **(1TTF, human)** | **K**  **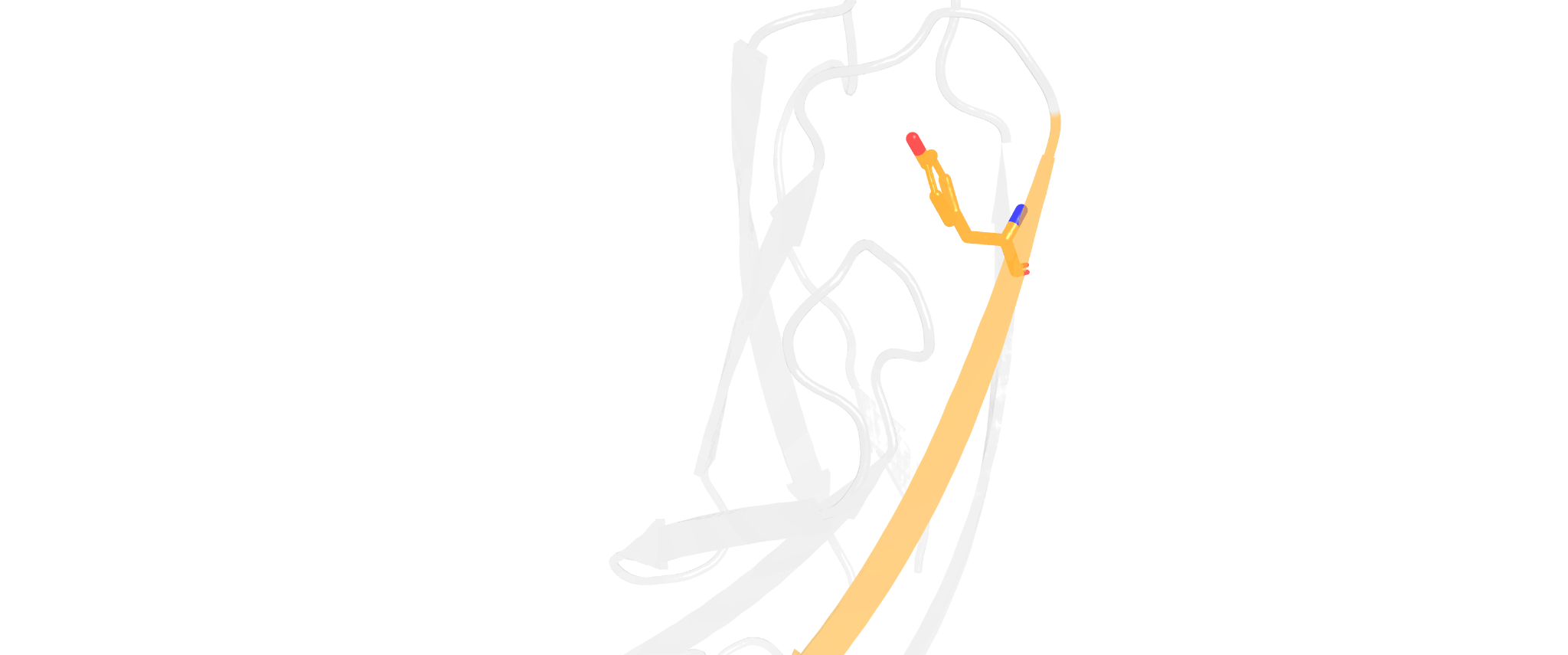**  **Polycystin-1**  **(1B4R, human)** | **L**  **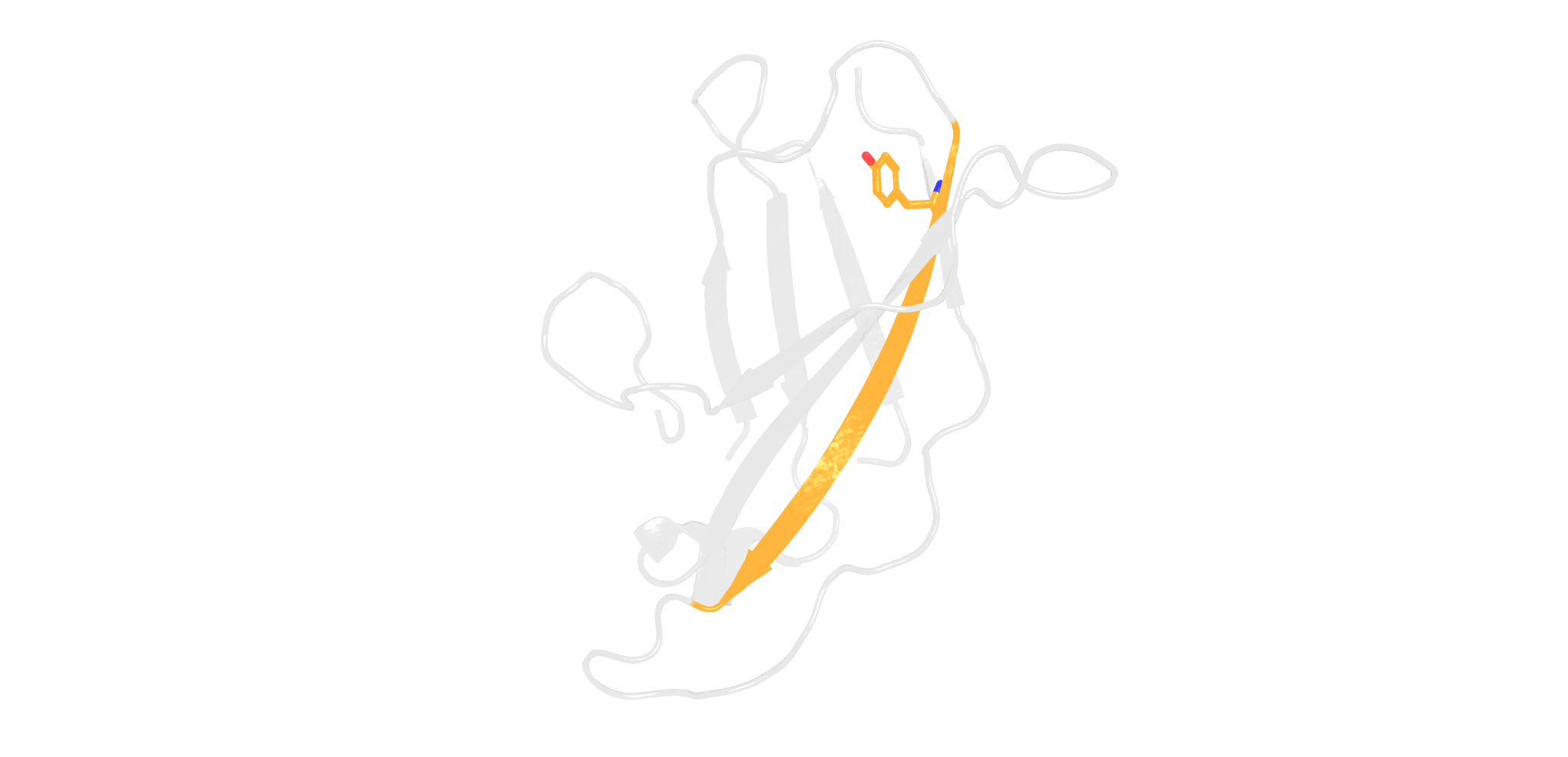**  **Thrombopoietin Receptor**  **(8G04, human)** |

**Fig B.** F strand Tyr (igs# 8548 or 8546) is highly conserved across many eukaryotic and bacterial Ig-like domains despite topo-structural variations in the fold. This Tyr appears to play similar roles by interacting with the EF loop, and may provide insights into the evolution of a number of proteins sharing the Ig-fold. It certainly points toward a key structural residue.

| **A- Contactin-2 (8AOY)**  **HorseShoe**  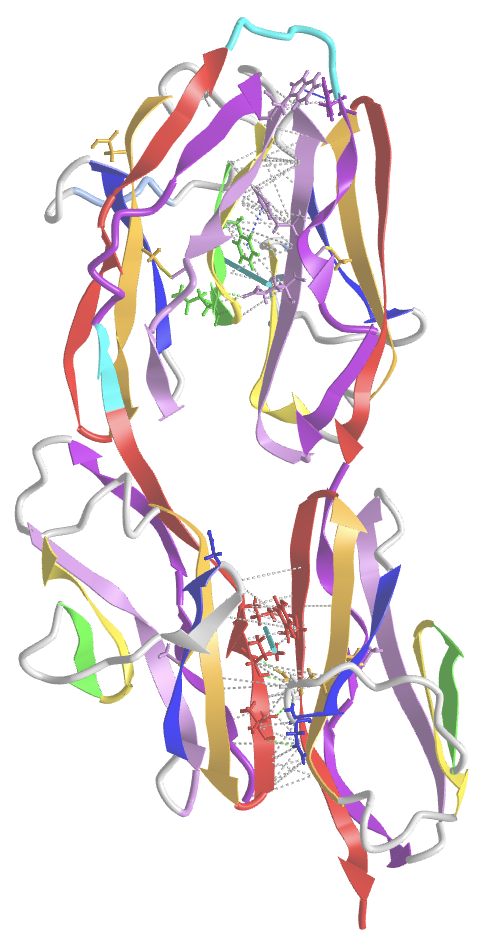 | **Contactin-2 (8AOY)**  **B- Ig1-Ig4**  **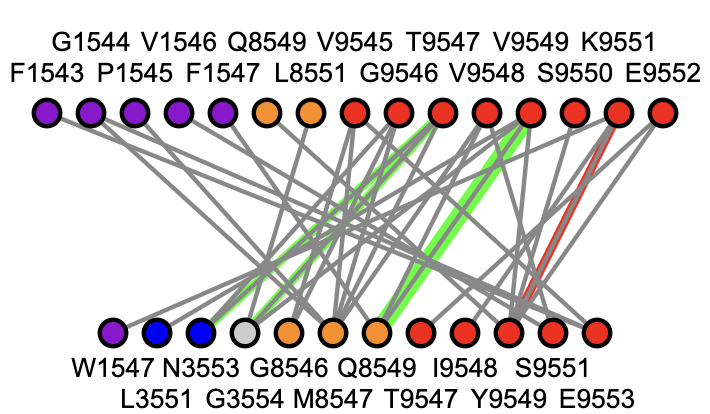**  **C- Ig2-Ig3**  **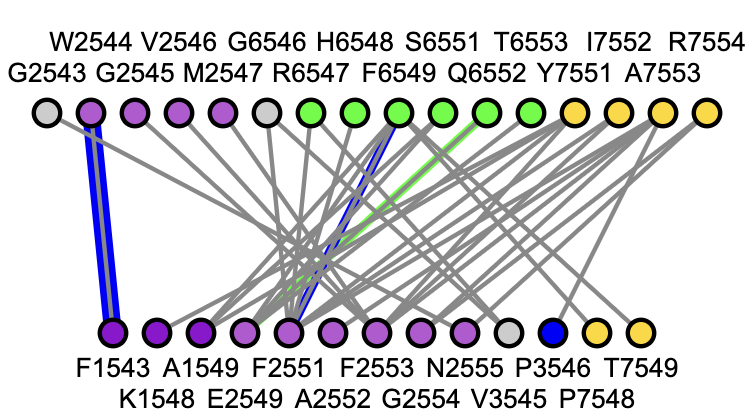**  **D- Ig1-Ig2 E- Ig3-Ig4**  **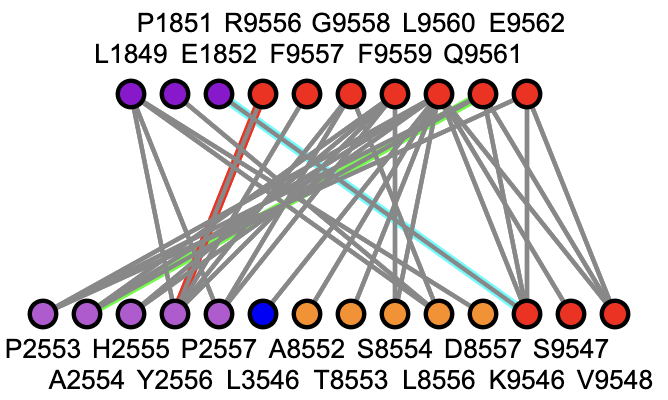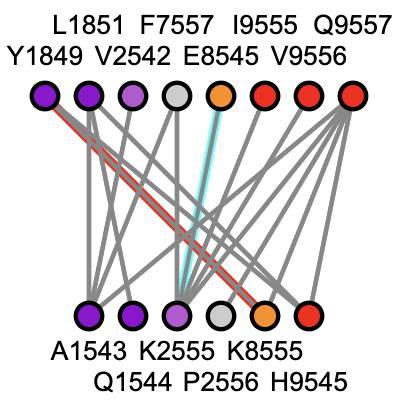** | **DSCAM (3DMK)**  **F- Ig1-Ig4**  **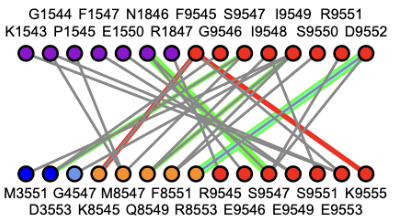**  **G- Ig2-Ig3**  **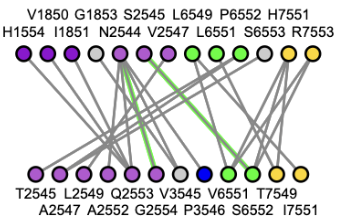**  **D- Ig1-Ig2 E- Ig3-Ig4**  **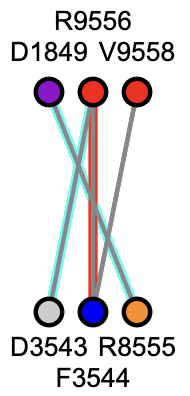 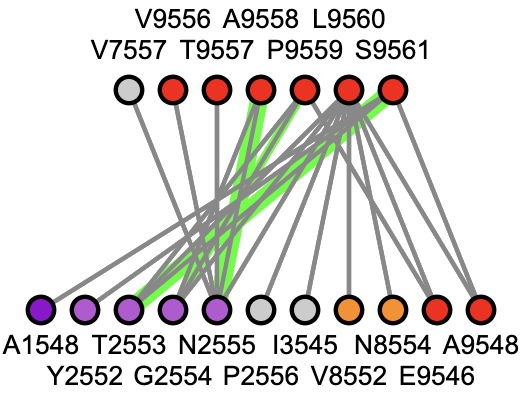** | **Common in 3DMK**  **J- Ig1-Ig4**  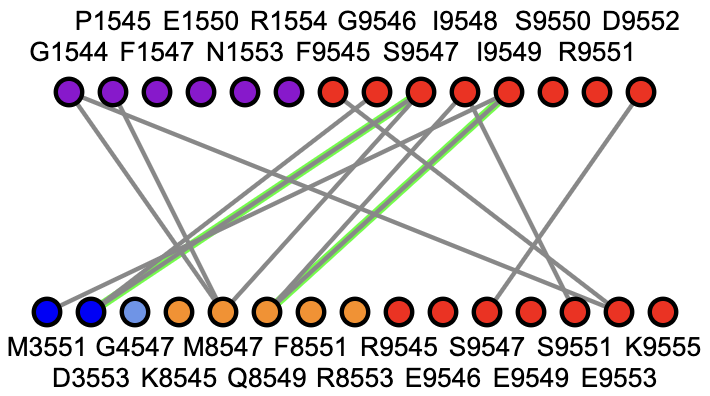  **K- Ig3-Ig4**  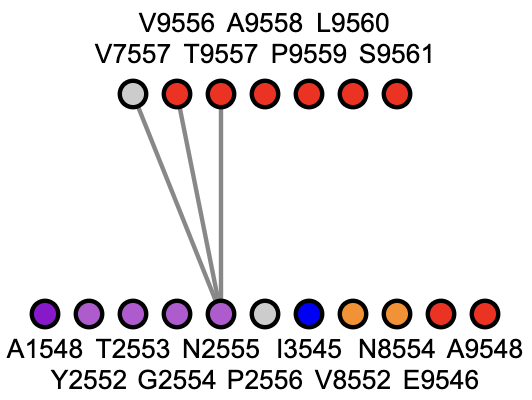 |
| --- | --- | --- | --- |

**Fig C.** Tertiary Intrachain interfaces in the Horseshoe superdomain formed by the four N-terminal residues in Ig-chains. This superdomain allows a certain level of plasticity observed in comparing contactin-2 and DSCAM. The RMSD is 4.9Å. A) Contactin-2 Horseshoe (Pdbid 8A0Y) shows an Ig1-Ig4 and a Ig2-Ig3 antiparallel interfaces. B) Ig1-Ig4 interactome in contactin-2. C) Ig2-Ig3 interactome in contactin-2. D) Ig1-Ig2 interactome in contactin-2. E) Ig3-Ig4 interactome in contactin-2. F) Ig1-Ig4 interactome in DSCAM. G) Ig2-Ig3 interactome in DSCAM. H) Ig1-Ig2 interactome in DSCAM. I) Ig3-Ig4 interactome in DSCAM. J) Common interactions in Ig1-Ig4. This pairwise interactome is more conserved than Ig2-Ig3 that is more plastic. K) Common interactions in Ig3-Ig4.

| 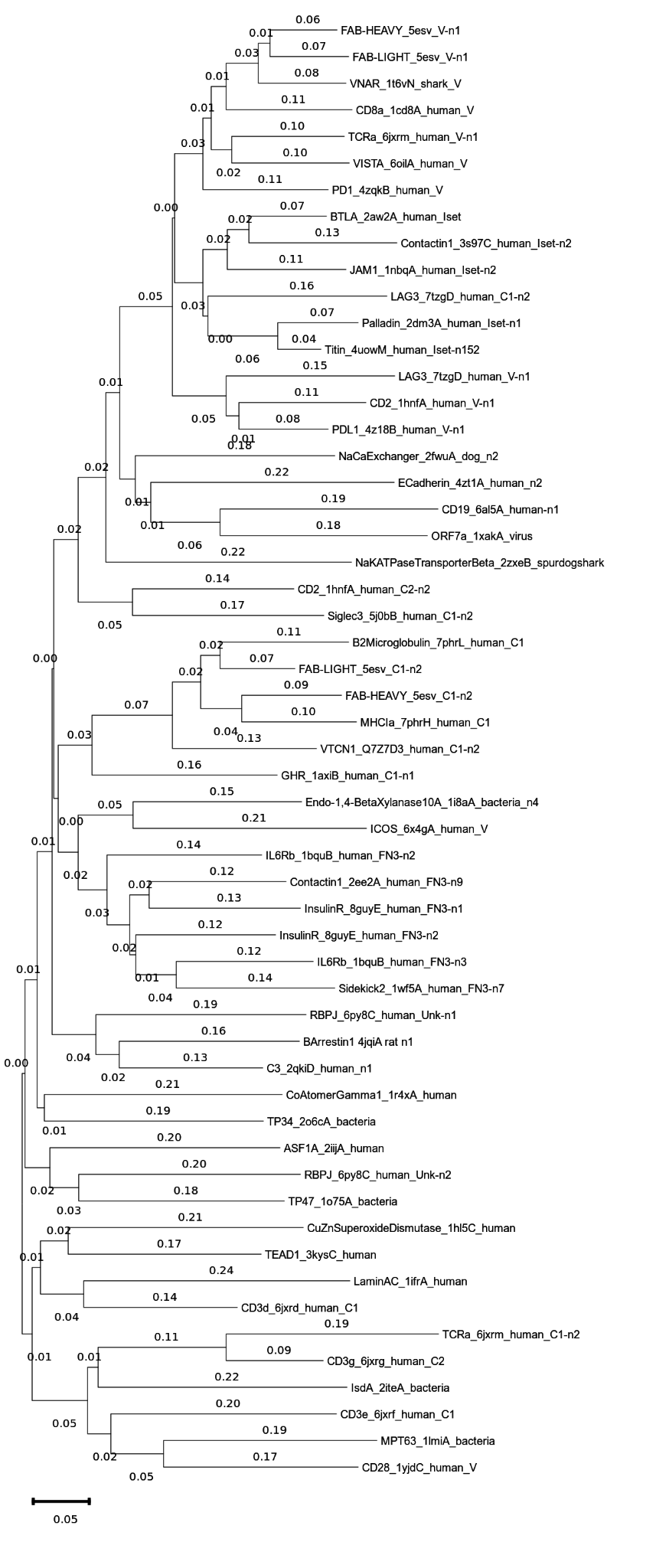 |
| --- |

**Fig D.** Clustering Ig templates using MEGA11. The graph shows the distances between templates. The corresponding TM-score is equal to 1 - distance.

**Table A.** Number of Heavy chain – Light chain contacts in VH:VL and CH1:CL interfaces in Fabs bound to SARS-CoV2 antigens or to a diverse set of antigens and present to more than 70% (or 90%) of the Fabs in each dataset.

| **VH:VL interface (parallel)** | SARS-CoV-2 antigen | Diverse antigens |
| --- | --- | --- |
| Total pairs (70% cutoff) | 16 | 14 |
| Symmetric pairs (70% cutoff) | 10 | 9 |
| Total pairs (90 % cutoff) | 7 | 6 |

| **CH1:CL interface (antiparallel)** | SARS-CoV-2 antigen | Diverse antigens |
| --- | --- | --- |
| Total pairs (70% cutoff) | 28 | 27 |
| Symmetric pairs (70% cutoff) | 3 | 3 |
| Total pairs (90 % cutoff) | 12 | 17 |

**Table B.**  VH:VL interactions of Fabs binding diverse antigens (70% cutoff).Red numbers represent symmetric contacts. Shaded cells represent five highly conserved contacts (90% cutoff) shared between the SARS-CoV-2 antigen binding dataset (Table 2 in manuscript) and this diverse antigen binding dataset. Bold contacts represent highly conserved hydrogen bonding contacts. Underlined contacts represent those that have been found previously [131]

| **VH igs#** | **VL igs#** | **#Fabs** | **% Fabs** | **contacts** | **hbonds** | **ionic** | **pi-stacking** | **pi-cation** |
| --- | --- | --- | --- | --- | --- | --- | --- | --- |
| 4547 | 9549 | 107 | 100 | 107 |  |  |  |  |
| 4549 | 9547 | 106 | 99 | 106 | 1 |  | 33 | 4 |
| **3553** | **3553** | 106 | 99 | 106 | **101** | 1 |  |  |
| 9549 | 4547 | 106 | 99 | 106 | 1 |  | 1 |  |
| 4547 | 8549 | 102 | 95 | 102 |  |  |  |  |
| 9550 | 4546 | 101 | 94 | 101 | 17 |  |  |  |
| 8549 | 4546 | 95 | 89 | 95 |  |  |  |  |
| 9549 | 3551 | 95 | 89 | 95 |  |  | 34 |  |
| **3553** | **8549** | 94 | 88 | 93 | 1 |  |  | 1 |
| **8549** | **3553** | 93 | 87 | 93 | 2 |  |  |  |
| 4549 | 9546 | 91 | 85 | 91 |  |  |  |  |
| **9546** | **3551** | 89 | 83 | 89 | **66** |  | 1 |  |
| 9547 | 4549 | 80 | 75 | 80 | 2 | 5 |  |  |
| 9546 | 4549 | 80 | 75 | 80 | 1 |  |  |  |

**Table C**. CH1:CL interactions of Fabs binding diverse antigens (70% cutoff). Red numbers represent symmetric contacts. Shaded cells represent twelve highly conserved contacts (90% cutoff) shared between SARS-CoV-2 antigen binding dataset (Table 3) and this diverse antigen binding dataset. Bold contacts show highly conserved hydrogen bonding and in purple for a highly conserved ionic contact.

| **CH1 igs#** | **CL igs#** | **#Fabs** | **% Fabs** | **contacts** | **hbonds** | **ionic** | **pi-stacking** | **pi-cation** |
| --- | --- | --- | --- | --- | --- | --- | --- | --- |
| 1549 | 1554 | 107 | 100 | 107 |  |  |  |  |
| 2547 | 1551 | 107 | 100 | 107 |  |  |  |  |
| 1549 | 1557 | 107 | 100 | 107 |  |  |  |  |
| 6553 | 7546 | 107 | 100 | 107 |  |  |  |  |
| 1551 | 1551 | 107 | 100 | 107 |  |  |  |  |
| 6553 | 7545 | 106 | 99 | 106 |  |  |  |  |
| 6553 | 2551 | 106 | 99 | 106 |  |  | 16 |  |
| 1550 | 1554 | 106 | 99 | 106 |  |  |  |  |
| 1552 | 1551 | 106 | 99 | 106 |  |  |  |  |
| **6551** | **7545** | 104 | 97 | 104 | **85** |  |  |  |
| 7549 | 2551 | 101 | 94 | 101 |  |  |  |  |
| 2553 | 1557 | 101 | 94 | 98 | 1 | 16 |  |  |
| 2553 | 2547 | 99 | 93 | 99 |  |  |  |  |
| 6553 | 7547 | 98 | 92 | 98 |  |  |  |  |
| **6554** | **6553** | 98 | 92 | 98 | **79** |  |  |  |
| 2547 | 1549 | 96 | 90 | 96 | 7 |  |  |  |
| 6551 | 2553 | 96 | 90 | 96 |  |  |  |  |
| 6558 | 6551 | 95 | 89 | 95 |  |  |  |  |
| 2551 | 2547 | 94 | 88 | 94 |  |  |  |  |
| 6556 | 6551 | 92 | 86 | 92 |  |  |  |  |
| 6551 | 2554 | 92 | 86 | 87 | 26 | 5 |  |  |
| **9550** | **1556** | 91 | 85 | 75 | 13 | **90** |  |  |
| 6554 | 6554 | 90 | 84 | 90 |  |  |  |  |
| 6553 | 6553 | 89 | 83 | 89 |  |  |  |  |
| 6553 | 6555 | 89 | 83 | 89 |  |  |  |  |
| 6556 | 6552 | 84 | 79 | 84 |  |  |  |  |
| 1551 | 2549 | 80 | 75 | 80 |  |  |  |  |

**Table D.** VH:VL common interaction pairs among Fabs binding the SARS-CoV-2 spike protein, Fabs binding a diverse set of antigens, and a pair of Fabs (PDB IDs: 3NGB, 4JPK) targeting the viral gp120 envelope glycoprotein. Red numbers represent symmetric contacts.

| **#igs VH** | **#igs VL** |
| --- | --- |
| 4547 | 8549 |
| 9550 | 4546 |
| 8549 | 4546 |
| 9546 | 4549 |
| 4549 | 9547 |
| 3553 | 3553 |
| 9549 | 3551 |
| 9547 | 4549 |
| 9546 | 3551 |
| 4547 | 9549 |
| 9549 | 4547 |

**Table E.** CH1:CL common interaction pairs among Fabs binding the SARS-CoV-2 spike protein, Fabs binding a diverse set of antigens, and a pair of Fabs (PDB IDs: 3NGB, 4JPK) targeting the viral gp120 envelope glycoprotein. Red numbers represent symmetric contacts.

| #igs CH1 | #igs CL |
| --- | --- |
| **6553** | **6553** |
| **6551** | **7545** |
| 1549 | 1557 |
| 1551 | 1551 |
| 2553 | 1557 |
| **6551** | **2553** |
| 2547 | 1551 |
| 1552 | 1551 |
| 1549 | 1554 |
| **6556** | **6551** |
| 9550 | 1556 |
| **6553** | **7546** |
| 1550 | 1554 |
| **6553** | **7547** |
| **6553** | **6555** |
| **6554** | **6553** |
| **6553** | **7545** |
| **6554** | **6554** |
| 2547 | 1549 |

**Table F.** CDR regions as defined by previous Ig numbering systems, mapped to IgStrand numbers.

| CDR | Kabat | Chothia | Martin | IMGT |
| --- | --- | --- | --- | --- |
| L1 | 24-34 \| 2551-3549 | 24-34 \|2551-3549 | 24-34 \| 2551-3549 | 27-32 \| 2554-3547 |
| L2 | 50-56 \| 4553-5552 | 50-56 \| 4553-5552 | 50-56 \| 4553-5552 | 50-52 \| 4553-5548 |
| L3 | 89-97 \| 8551-9548 | 89-97 \| 8551-9548 | 89-97 \| 8551-9548 | 89-97 \| 8551-9548 |
| H1 | 31-35 \| 3543-3549 | 26-32 \| 2554-3546 | 26-35 \| 2554-3549 | 26-33 \| 2554-3549 |
| H2 | 50-65 \| 4552-6546 | 52-56 \| 4554-5547 | 50-58 \| 4552-5549 | 51-57 \| 4553-5548 |
| H3 | 95-102 \| 8553-9548 | 95-102 \| 8553-9548 | 95-102 \| 8553-9548 | 93-102 \| 8551-9548 |
