# Supplementary material for: IgStrand: A universal residue numbering scheme for the immunoglobulin-fold (Ig-fold) to study Ig-proteomes and Ig-interactomes
Source: PLoS Comput Biol. 2025 Apr 14;21(4):e1012813. doi: 10.1371/journal.pcbi.1012813 (PMC12051499; doi:10.1371/journal.pcbi.1012813)
Supplement: Attachment — Submitted filename: PCOMBBIOL-D-24-01553_Response-to-Reviewers.pdf [file pcbi.1012813.s015.pdf]

We would like to thank the reviewers for their helpful comments that have helped us in improving the manuscript. Here are our point-by-point responses to those comments. The reviewer comments are listed below in *italics* followed by our responses as normal text. In response to the comments, when changes were made to the manuscript, the paragraphs where changes were made are highlighted in blue in the revised manuscript. (Apart from the antibody analysis section requested by reviewer #2, the changes are not as extensive as the blue text may suggest since it is the result of the rearrangement of paragraphs shifted from the Results and discussion section to the introduction, as rightly asked by reviewer #1).

Reviewer #1:

[Rev1C0] The authors provide an updated numbering scheme to identify Ig structural proteomes and interactomes, which allow researchers to communicate about protein structures and function in a standardized fashion. The numbering scheme is innovative in that it does not solely rely on sequence alignments but incorporates the structure and folding patterns. This new scheme is human readable as well as machine readable, allowing for new machine learning algorithms to be implemented.

[Response_Rev1C0] We thank the reviewer for their appreciation of some key features of our IgStrand numbering scheme.

Comments:

[Rev1C1] The Introduction is a nice review on Ig folds but doesn't mention the previous numbering schemes until the Results and Discussion section. The information becomes a bit redundant. I would suggest moving some of the discussion in the "Results and Discussion" to the Introduction section to differentiate between older numbering schemes and the contribution of this current work. The "Definition of the IgStrand numbering" section on page 15 seems to be the start of the Results section.

[Response_Rev1C1] This is a great point. We have moved the first few sections of the "Results and Discussion" section to the end of the "Introduction" section. Now the "Results and Discussion" section starts as suggested by the reviewer.

[Rev1C2] Terms are defined later in the article after they are used instead of the other way around. An example is the description of the A strand split on page 12, when it is mentioned on page 6.

[Response_Rev1C2] We have removed the initial reference to A strand split and tried to catch similar instances.

[Rev1C3] Page 4, last paragraph, the second sentence is a long incomplete sentence with several references making it difficult to read.

[Response_Rev1C3] We have broken up the long sentence and fixed it to improve its readability.

[Rev1C4] Page 15, the scheme is described as using 4 digits, but the 3rd and 4th digit are not discussed until a couple of sections later. A brief mention of all four in the beginning would help with the transitions.

[Response_Rev1C4] This was a good point and we have fixed it by introducing all 4 digits together.

[Rev1C5] Page 6, last paragraph, first sentence: “The immunoglobulin fold is both one and many” is a bit confusing.

[Response_Rev1C5] That sentence and paragraph has been fixed for clarity.

[Rev1C6] Figure 3 legend. What does the # represent in the figure? CD4, ICOS, PD-L2, and VNAR domains are mentioned in the legend but not shown in the figure. This should remain in the main text of the article.

[Response_Rev1C6] The # represents the anchor residue for each strand that will be numbered xx50. This is now included in the revised figure legend. Also, the text about CD4, ICOS, PD-L2, and VNAR has been moved to the text where Figure 3 was introduced.

[Rev1C7] Table 1. The font should be bigger. It is difficult to read.

[Response_Rev1C7] The table 1 has been updated to show only the key columns making the text easier to read.

[Rev1C8] Page 30, last paragraph, first sentence: The word “least” is written twice.

[Response_Rev1C8] This has been fixed.

Reviewer #2:

[Rev2C0] In this work, authors have developed a universal numbering system IgStrand numbering that enables positional comparison of the Immunoglobulin fold (Ig-fold). Ig-fold belong to the Ig Superfamily and is the most populous domain in human genome accounting for 2% of the coding genes. Ig domains are ubiquitous, playing important roles in cellular communications and Immunoglobulin variable domains are the most studied Ig domains.
Ig domains are made of 7-9 strands that make a sandwich of two β sheets. IgStrand numbering (ij50) consists of 4 digits and assigns number 50 to the “anchor residue” in each of the 9 main canonical Ig domain strands: A, B, C, C’, C”, D, E, F and G. Anchor residues are assigned based on both structural considerations and sequence conservation. Digit “i” spans from 1-9 and represents the canonical strands with their respective order. Digit “j” is usually 5, but strand insertions and splits can be assigned with j+1 or j-1. Residues are number linearly ij50-n or ij50+n if they come before or after the anchor residue.
Using their IgStrand numbering, authors present diverse set of Ig-folds such as Arrestin, Lamin, Transcription factors and Ig-domains in viruses shining light on their structural diversity and variations. Then authors explore Ig-Ig domain interfaces in library of Fab fragments that target SARS-CoV-2 spike protein identifying 361 + 213 unique interacting residue pairs for VH|VL and CH1|CL respectively. Finally, authors explore more complex Ig-chain interfaces such as Tandem IgV1-IgV2 and Ig-Horseshoe domains.

Comments.

• Authors explore an interesting topic, and they managed to come up with a sequence numbering system for the entire Ig Superfamily.
• Authors were able to present their IgStrand numbering in a clear and easy to digest way with nice visual presentation of anchor residues (Figure 6).
• IgStrand numbering is linear and easy to read for humans making comparison of Ig-folds with numerous topostructural variations easier. Furthermore, machine readability of IgStrand numbering could enable comparison of large dataset of Ig domains and quaternary interfaces.

[Response_Rev2C0] We thank the reviewer for an excellent summary of the IgStrand numbering scheme. They have captured many of the strengths of this scheme.

Critical comments:

[Rev2C1] • Figure 3 a. b. c. is lowercase, but other figures are capitalized such as Figure 5 A, B, C.

[Response_Rev2C1] This exception for Figure 3 was done to avoid any confusion with the strands A through G that are being highlighted in this figure.

[Rev2C2] • I liked the fact that Authors made IgStrand numbering linear by reserving j for strand insertions and splits (Figure 18 B). A-, and C- are numbered j = 5-1 and A+ and G+ strands are numbered j = 5+1. Although it was mentioned in Page 16 that A' is a special case and its anchor was numbered j = 5+3 (1850). However, I did not understand why A strand skips j = 7. Was it reserved for something? I think authors should clarify that.

[Response_Rev2C2] The Ig domain in protein p53 is an example where an A+ strand is seen. j=7 was skipped to allow for the possibility of an A++ strand before A' strand. This has been clarified in the IgStrand numbering definition section.

[Rev2C3] • In Page 30, Authors compare library of 258 Fab fragments. They found 361 unique interacting pairs for VH|VL and they found 231unique interacting pairs for CH1|CL. Since all of these antibodies target the same protein, it is expected to see common interacting pairs for VH|VL even if they might be targeting different epitopes within the same protein. However, considering CH and CL domains are constant, one could expect more common pairs for CH1|CL relative to VH|VL. Authors should further explore this by comparing VH|VL and CH1|CL in library of random Fabs targeting different antigens (ideally, similar size library) to see if common antigen target increases the common residue pairs in VH|VL.

[Response_Rev2C3] We thank the reviewer for this comment and proposed experiments. To make the analysis robust, we filtered our structural dataset to select high resolution 107 structures with Fabs bound to SARS-CoV-2 antigen and added a second set of 107 structures with Fabs bound to a **diverse antigen set.** The VH|VL and CH1|CL contact analysis was carried out for these two sets independently, where we focused on identifying common interacting pairs of residues (found in at least 70% of the structures). The results (Figure 13, Table 2, Table 3) and corresponding discussion has replaced our

previous section on this. The supplementary table S1 shown below summarizes the results that the reviewer will find very interesting.

Table S1: Number of Heavy chain - Light chain contacts in VH:VL and CH:CL interface in Fab domains bound to SARS-CoV2 antigens or a diverse set of antigens.

**VH:VL interface (parallel)**	SARS-CoV-2 antigen	Diverse antigens
Total contact pairs (70% cutoff)	16	14
Symmetric pairs (70% cutoff)	10	9
90 % pairs	7	6

**CH1:CL interface (antiparallel)**	SARS-CoV-2 antigen	Diverse antigens
Total contact pairs (70% cutoff)	28	27
Symmetric pairs (70% cutoff)	3	3
90 % pairs	12	17

The VH|VL and CH1|CL common interactomes in the two sets are consistent, and the small variations are mentioned in the text. This two-sets comparison led us to start a more in-depth analysis that should be the focus of a dedicated paper on the whole set of antibodies in the PDB. We have also revised our criteria for identifying residue contacts for consistency between structures that contain hydrogens and those that do not in the PDB. We are not citing all individual pairs that may occur in any of the individual VH|VL or CH1|CL interfaces, but focus on the "common" interactome for a number of reasons, among them accuracy since the main aim here is in demonstrating the use and universality of the IgStrand numbering, while the quality and accuracy can, should, and will be improved in the work that we are planning. We have nonetheless improved the contact analysis to be agnostic on the presence or not of hydrogens in PDB structures and give more consistent interacting pairs across these dataset. Future contact analysis should rely on regularized structures and more stringent criteria for different types of interactions. A future full scale study should improve contact criteria as well as structure quality with a) a bigger dataset of antibody structures, b) addition of hydrogens to all structures, c) regularization (energy minimization) of structures and optimization of sidechain rotamers to locally optimize hydrogen bonding, ionic, or van der Waals contacts, and for a diverse subset of cases e) molecular dynamics simulations. In a future study an analysis of the full Fab conformations should be envisioned. In searching the literature for relevant studies, we have found a reference (Sheng et al. 2022) that had examples where drastic conformational changes occur due to somatic hypermutations between VCR01 and VCR01gl with drastic conformational change. We have added a comparison on VH|VL interactome that counts 11 common contacts, and CH1|CL interactome that counts 19 common contacts conserved. This small scale analysis shows that we also have to consider V|C plasticity involving their interfaces and Hinges in the future Fab analysis.

[Rev2C4] Overall, Authors present interesting and impactful study that can help other researchers in the field. They give enough background to highlight the novelty in their work and their explanation is easy to digest. However, I believe the experiment suggested above is critical to understand the result of IgVH-IgVL and IgCH1-IgCL interactome experiment. Therefore, I believe Authors should address this question along with other two minor comments before publication.

[Response_Rev2C4] The experiment proposed by the reviewer described earlier was indeed successful and has inspired us for a future in-depth study on antibodies, that will also serve the study of non-antibody chains, especially cell surface receptors that are a big part of our research at both the tertiary and quaternary level. We hope that we have addressed the reviewer's comments and we thank the reviewer for helping us improve this study and for raising priorities for future studies on our end.

As observed by the reviewer, our intention was effectively to help researchers in the field, by giving a tool to the community to unravel observations that are just too difficult to make at scale and compare without a universal numbering system. We thought and think that a myriad of structural observations on folding and on protein-protein interactions can be made by many researchers working on many Ig-based proteins, and we hope our contribution may help in that regard, and above all in the ability to share these (numbered) observations. Naturally, some improvement can still be envisioned on numbering, especially for multi-Ig chains (Hinges) but also for strand insertions in Ig-extended domains, and possibly in loops. We will work on all of these fronts, hopefully with a positive and steady community feedback.
