# Supplementary material for: IgStrand: A universal residue numbering scheme for the immunoglobulin-fold (Ig-fold) to study Ig-proteomes and Ig-interactomes
Source: PLoS Comput Biol. 2025 Apr 14;21(4):e1012813. doi: 10.1371/journal.pcbi.1012813 (PMC12051499; doi:10.1371/journal.pcbi.1012813)
Supplement: Attachment — Submitted filename: Review, comments for the Authors.docx [file pcbi.1012813.s013.docx]

In this work, authors have developed a universal numbering system **IgStrand numbering** that enables positional comparison of the Immunoglobulin fold (Ig-fold). Ig-fold belong to the Ig Superfamily and is the most populous domain in human genome accounting for 2% of the coding genes. Ig domains are ubiquitous, playing important roles in cellular communications and Immunoglobulin variable domains are the most studied Ig domains.

Ig domains are made of 7-9 strands that make a sandwich of two β sheets. IgStrand numbering (ij50) consists of 4 digits and assigns number 50 to the “anchor residue” in each of the 9 main canonical Ig domain strands: A, B, C, C’, C’’, D, E, F and G. Anchor residues are assigned based on both structural considerations and sequence conservation. Digit “i” spans from 1-9 and represents the canonical strands with their respective order. Digit “j” is usually 5, but strand insertions and splits can be assigned with j+1 or j-1. Residues are number linearly ij50-n or ij50+n if they come before or after the anchor residue.

Using their IgStrand numbering, authors present diverse set of Ig-folds such as Arrestin, Lamin, Transcription factors and Ig-domains in viruses shining light on their structural diversity and variations. Then authors explore Ig-Ig domain interfaces in library of Fab fragments that target SARS-CoV-2 spike protein identifying 361 + 213 unique interacting residue pairs for VH|VL and CH1|CL respectively. Finally, authors explore more complex Ig-chain interfaces such as Tandem IgV1-IgV2 and Ig-Horseshoe domains.

Comments.

- Authors explore an interesting topic, and they managed to come up with a sequence numbering system for the entire Ig Superfamily.
- Authors were able to present their IgStrand numbering in a clear and easy to digest way with nice visual presentation of anchor residues (Figure 6).
- IgStrand numbering is linear and easy to read for humans making comparison of Ig-folds with numerous topostructural variations easier. Furthermore, machine readability of IgStrand numbering could enable comparison of large dataset of Ig domains and quaternary interfaces.

Critical comments:

- Figure 3 a. b. c. is lowercase, but other figures are capitalized such as Figure 5 A, B, C.
- I liked the fact that Authors made IgStrand numbering linear by reserving j for strand insertions and splits (Figure 18 B). A-, and C- are numbered j = 5-1 and A+ and G+ strands are numbered j = 5+1. Although it was mentioned in Page 16 that A’ is a special case and its anchor was numbered j = 5+3 (1850). However, I did not understand why A strand skips j = 7. Was it reserved for something? I think authors should clarify that.
- In Page 30, Authors compare library of 258 Fab fragments. They found 361 unique interacting pairs for VH|VL and they found 231unique interacting pairs for CH1|CL. Since all of these antibodies target the same protein, it is expected to see common interacting pairs for VH|VL even if they might be targeting different epitopes within the same protein. However, considering CH and CL domains are constant, one could expect more common pairs for CH1|CL relative to VH|VL. Authors should further explore this by comparing VH|VL and CH1|CL in library of random Fabs targeting different antigens (ideally, similar size library) to see if common antigen target increases the common residue pairs in VH|VL.

Overall, Authors present interesting and impactful study that can help other researchers in the field. They give enough background to highlight the novelty in their work and their explanation is easy to digest. However, I believe the experiment suggested above is critical to understand the result of IgVH-IgVL and IgCH1-IgCL interactome experiment. Therefore, I believe Authors should address this question along with other two minor comments before publication.
